# Pharmacokinetics, Biodistribution, and Biosafety of PEGylated Gold Nanoparticles In Vivo

**DOI:** 10.3390/nano11071702

**Published:** 2021-06-28

**Authors:** Katarina Kozics, Monika Sramkova, Kristina Kopecka, Patricia Begerova, Alena Manova, Zora Krivosikova, Zuzana Sevcikova, Aurelia Liskova, Eva Rollerova, Tibor Dubaj, Victor Puntes, Ladislava Wsolova, Peter Simon, Jana Tulinska, Alena Gabelova

**Affiliations:** 1 Department of Nanobiology, Cancer Research Institute, Biomedical Research Center SAS, 845 05 Bratislava, Slovakia; monika.sramkova@savba.sk (M.S.); kristina.kopecka@savba.sk (K.K.); patricia.pechacova@gmail.com (P.B.); alena.gabelova@savba.sk (A.G.); 2 Institute of Analytical Chemistry, Slovak University of Technology in Bratislava, 812 37 Bratislava, Slovakia; alena.manova@stuba.sk; 3 Department of Clinical and Experimental Pharmacotherapy, Slovak Medical University in Bratislava, 833 03 Bratislava, Slovakia; zora.krivosikova@szu.sk; 4 Department of Morphological Disciplines, University of Veterinary Medicine and Pharmacy in Kosice, 041 81 Kosice, Slovakia; zuzana.sevcikova@uvlf.sk; 5 Laboratory of Immunotoxicology, Slovak Medical University in Bratislava, 833 03 Bratislava, Slovakia; aurelia.liskova@szu.sk (A.L.); jana.tulinska@szu.sk (J.T.); 6 Laboratory of Toxicology, Slovak Medical University in Bratislava, 833 03 Bratislava, Slovakia; eva.rollerova@szu.sk (E.R.); ladislava.wsolova@szu.sk (L.W.); 7 Institute of Physical Chemistry and Chemical Physics, Slovak University of Technology in Bratislava, 812 37 Bratislava, Slovakia; tibor.dubaj@stuba.sk (T.D.); peter.simon@stuba.sk (P.S.); 8 Catalan Institute of Nanotechnology, UAB Campus, 08193 Bellaterra, Barcelona, Spain; victor.puntes@icn2.cat

**Keywords:** gold nanoparticles, rats, pharmacokinetics, biodistribution, histopathology, clinical chemistry, hematology

## Abstract

Despite the obvious advantages of gold nanoparticles for biomedical applications, controversial and incomplete toxicological data hamper their widespread use. Here, we present the results from an in vivo toxicity study using gold nanoparticles coated with polyethylene glycol (PEG-AuNPs). The pharmacokinetics and biodistribution of PEG-AuNPs were examined in the rat’s liver, lung, spleen, and kidney after a single i.v. injection (0.7 mg/kg) at different time intervals. PEG-AuNPs had a relatively long blood circulation time and accumulated primarily in the liver and spleen, where they remained for up to 28 days after administration. Increased cytoplasmic vacuolation in hepatocytes 24 h and 7 days after PEG-AuNPs exposure and apoptotic-like cells in white splenic pulp 24 h after administration has been detected, however, 28 days post-exposure were no longer observed. In contrast, at this time point, we identified significant changes in lipid metabolism, altered levels of liver injury markers, and elevated monocyte count, but without marked biological relevance. In blood cells, no DNA damage was present in any of the studied time intervals, with the exception of DNA breakage transiently detected in primary kidney cells 4 h post-injection. Our results indicate that the tissue accumulation of PEG-AuNPs might result in late toxic effects.

## 1. Introduction

The outstanding physicochemical properties, well-established synthetic procedures, and easy surface modifications make gold nanoparticles (AuNPs) an emerging platform for a wide range of pharmaceutical and biomedical applications. The favorable optical behavior, tunable and surface plasmon resonance properties predispose AuNPs utilization, especially in ultrasensitive image-based diagnostic techniques such as photoimaging including photoacoustic imaging and computed tomography (CT) [1]. Furthermore, AuNPs are a valuable tool for biosensors, immunoassays, photothermal, photodynamic as well as targeted drug delivery and gene therapy [2]. The use of AuNPs is not limited only to the field of oncology. Other prospective therapeutic approaches have also been reported, such as treatment of Alzheimer’s and Parkinson’s diseases, HIV/AIDS, obesity and diabetes, tissue engineering, and ophthalmology [3].

Despite the intensive research and increased development and production of new gold-based nanopharmaceuticals, only a few of them have been investigated in clinical trials to date [2], and none has been approved by the US Food and Drug Administration (FDA) or European Medicines Agency (EMA) for clinical use [3]. 

Gold in its native bulk form has been considered a biocompatible, non-toxic, and biologically inert metal. Colloidal gold has been used for decades as a drug for rheumatoid arthritis (e.g., auranofin, allochrysin, or sanochrysin), also known as chrysotherapy [4]. However, after the transition from bulk to nano-scale material, gold acquires new unique physicochemical properties, including high reactivity with biological fluids resulting in protein corona formation [5]. Therefore, the potential of AuNPs to induce toxic effects on human health remains to be deciphered. Numerous in vitro and in vivo studies investigating AuNPs toxicity have been carried out during the past years but the obtained results are inconsistent. While some of them have shown that AuNPs are not toxic, many others contradict this statement [6,7]. Physicochemical characteristics such as size, shape, surface chemistry and charge, routes of administration, applied doses, exposure time, biodistribution, and accumulation in organs substantially affect the behavior of AuNPs in biological models, determine their interactions at the cellular and molecular levels, and consequently influence their toxic effects [8,9]. 

The majority of risk assessment studies have been performed in vitro using different cell lines. Such types of experiments provide valuable information about the mechanisms of AuNPs uptake and potential interactions at the cellular and molecular levels as the experimental conditions are strictly defined. However, in vitro experiments cannot fully model the complexity of the organism and behavior of AuNPs in different organs and their interaction with various cell types, which needs to be taken into account when extrapolating the potential health risk to humans. The number of published in vivo studies is limited and more data are needed to comprehensively assess the biosafety of AuNPs. To overcome this knowledge gap, our study aims to investigate the pharmacokinetics, biodistribution, and potential toxic effects of PEG-AuNPs after single intravenous (i.v.) administration. Rats were injected with a single dose of 0.7 mg/kg body weight and the effect of PEG-AuNPs was examined 1 h, 4 h, 24 h, 7 days, and 28 days post-exposure. AuNPs are considered to be relatively difficult to biodegrade, they remain accumulated in organs/tissues for an extended period or permanently [10]. Their long-term unintended retention time in the organism due to poor excretion raises further safety issues [11]. Studies employing lower exposure doses and examining biodistribution and biological effects for longer periods, better resembling human lifetime exposure, are therefore needed as they can contribute to a better understanding of the potential impacts of AuNPs on human health. 

## 2. Material and Methods

### 2.1. Chemicals

Ethidium bromide (EtBr, CAS No. 1239-45-8,), low-melting-point agarose (LMP, CAS No. 9012-36-6), normal-melting-point agarose (NMP, CAS No. 9012-36-6), Triton X-100 (CAS No. 9002-93), HEPES (CAS No. 7365-45-9), and collagenase (CAS No. 9001-12-1) were purchased from Merck (Merck KGaA, Darmstadt, Germany), and formamidopyrimidine-DNA glycosylase/AP nuclease (Fpg) was purchased from New England Biolabs (Ipswich, MA, USA). Culture media, fetal bovine serum (FBS), antibiotics, and other chemicals used for cell cultivation were purchased from Thermo Fisher Scientific (Waltham, MA, USA). All other chemicals and solvents were of analytical grade from commercial suppliers.

### 2.2. Gold Nanoparticles

**Figure 1:**
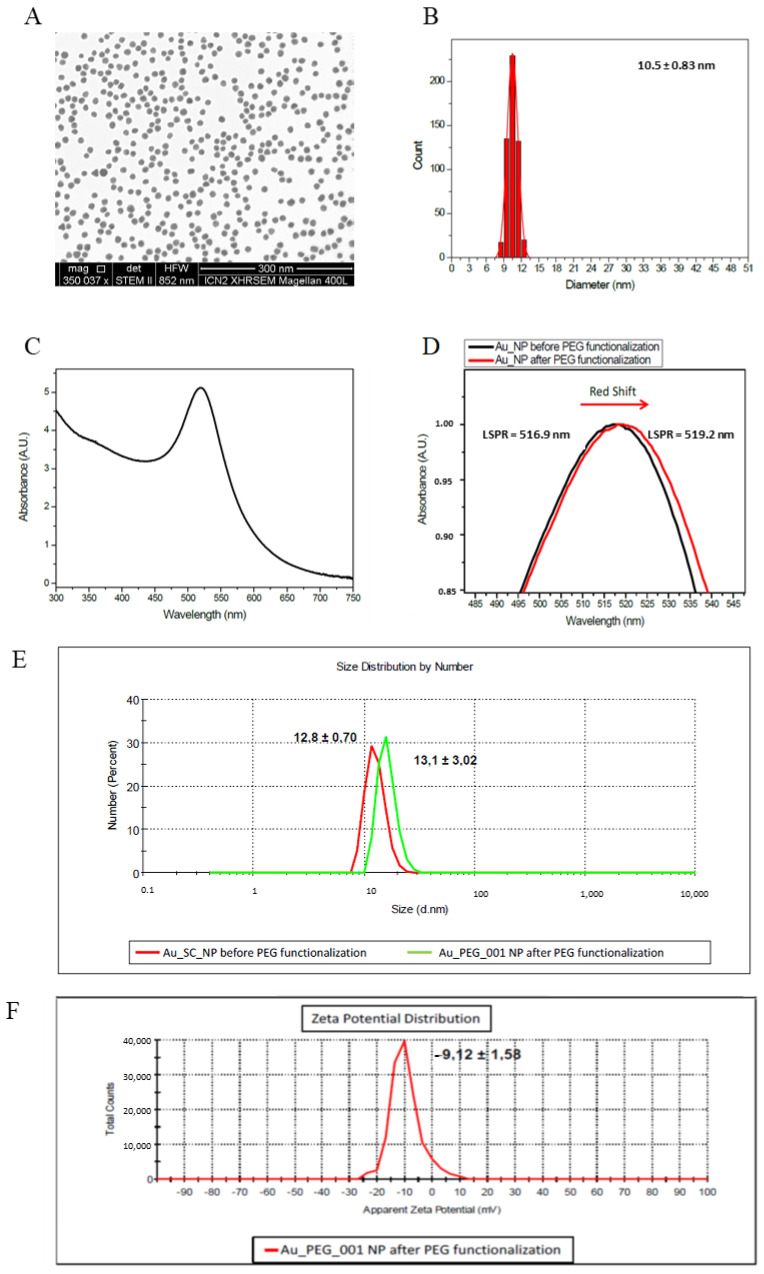
Basic characteristics of PEG-AuNPs. (**A**) TEM image of PEG-AuNPs; the bar represents 300 nm; (**B**) corresponding histograms of size distribution, (**C**) UV-vis spectra of the colloidal AuNPs suspension; (**D**) localized surface plasmon resonance (LSPR) of PEG-AuNPs; (**E**) hydrodynamic diameters measured by dynamic light scattering (DLS) for AuNPs and PEG-AuNPs, (**F**) zeta potential distribution of PEG-AuNPs.

PEG-AuNPs were kindly provided by Prof. Victor M. Puntes, Catalan Institute of Nanoscience and Nanotechnology, ICN2, Spain. Basic physicochemical characteristics of PEG-AuNPs are presented in Figure 1 and summarized in Table 1. PEG-AuNPs were dispersed in sterile MilliQ-water. The analytical screening revealed no detectable levels of endotoxin contamination within the PEG-AuNPs solution (<0.25 EU/mL).

### 2.3. Animals

The experiment was carried out on six to eight-week-old male Wistar rats obtained from the Department of Toxicology and Laboratory Animal Breeding (Dobra Voda, Slovakia) following the institutional guidelines under the approved protocols. Rats were housed in humidity and temperature-controlled ventilated cages on a 12 h day/night cycle with a rodent diet and water provided ad libitum. The in vivo experiment was approved by the Institutional Ethics Committee and by the national competent authority (State Veterinary and Food Administration of the Slovak Republic), under registration no. Ro 2807/12–221. The study was performed in compliance with the Directive 2010/63/EU and Regulation 377/2012 on the protection of animals used for scientific purposes in the approved animal facility (license no. SK PC 14011).

### 2.4. In Vivo Study Design

Rats were randomly divided into two experimental groups: (i) control group and (ii) PEG-AuNPs group. Before injection, each animal was weighed and the volume of a colloidal suspension of PEG-AuNPs was adjusted to apply the same dose to individual rats per group. The animals were divided into 2 groups (control, PEG-AuNPs). Each group comprised of 4–8 animals according to time intervals (1 h, 4 h, 24 h; 7 days, 28 days). There were 4 control rats and 5 injected rats for 1 h, 4 h, 24 h, 7 days, and 8 animals for the 28-day time point. The administered dose of PEG-AuNPs (0.7 mg/kg) was within the range of those found in the literature [12]. Animals (−210–230 g; *n* = 5–8 per group) were anaesthetized with intraperitoneal injection (i.p.) of Zoletil 100 (30 mg/kg) in combination with Xylariem (15 mg/kg) prior to i.v. injection of PEG-AuNPs. A single dose of PEG-AuNPs (0.7 mg/kg) was slowly administered into the rat tail vein. Animals in the control group were injected with phosphate-buffered saline (PBS, control for PEG-AuNPs, <400 µL/animal). At 1 h, 4 h, 24 h, 7 days, and 28 days after PEG-AuNPs injection and overnight fasting, the cardiac puncture for blood withdrawal was performed, while the animals were under anesthesia, which led to the death of the animal. Blood and organs/tissues, including the liver, lungs, spleen, kidneys, were collected and processed immediately or stored according to further analysis/measurement. 

### 2.5. Quantification of PEG-AuNPs in the Organism

The internalized amount of gold in the blood and organs at particular time-point intervals was quantified by graphite furnace atomic absorption spectrometry (GFAAS) using a high-resolution atomic absorption spectrometer AA 700 (Analytic Jena AG, Jena, Germany). The digestion of whole blood and tissue was realized by high-pressure microwave digestion with a mixture of HNO_3_-HCl. The measurements were performed using pyrolytically coated graphite tubes with an integrated PIN platform (Analytik Jena Part No 407-A81.026). The limit of detection (LOD) was determined to be lower than 0.125 ppb (μg/L). 

To compare the efficacy of PEG-AuNPs’ accumulation in the blood and individual organs throughout the study, all data calculations were expressed as the percentage of injected dose.

### 2.6. Pharmacokinetics of PEG-AuNPs

The time course of PEG-AuNPs concentration in blood was analyzed using a non-PBPK two-compartment model with elimination from the central compartment. In this case, the peripheral compartment did not correspond to any specific tissue or organ and the whole analysis was based solely on data from the central compartment (blood). The concentration in the central compartment follows a bi-exponential function

(1) C(t)=Aexp(−αt)+Bexp(−βt),

where A, B, α, and β are composite parameters derived from first-order rate constants of PEG-AuNPs translocation between central and peripheral compartment ($K_{12}$ and $K_{21}$), elimination rate constant ($K_{10}$), and initial concentration, C(0). The bi-exponential function was fitted to data using the non-linear least-squares method (OriginPro 9.1, OriginLab, MA, USA). Using the transformed parameters, the following pharmacokinetic descriptors can be calculated [13].

(2) $$t_{1/2\alpha }=\frac{\ln2}{\alpha }$$

(3) $$t_{1/2\beta }=\frac{\ln2}{\beta }$$

(4) $${\left(\mathrm{AUC}\right)}_{0-\infty }=\int_{0}^{\infty }C\left(t\right)\;dt=\frac{A}{\alpha }+\frac{B}{\beta }$$

(5) $$V_{d}=\frac{\mathrm{Dose}}{C\left(0\right)}$$

(6) $$t_{\max}=\frac{\ln\alpha -\ln\beta }{\alpha -\beta }$$

(7) $$C_{\max}=\frac{\mathrm{Dose}\cdot K_{12}\;}{V_{d}\left(\beta -\alpha \right)}\left[\exp\left(-\alpha t_{\max}\right)-\exp\left(-\beta t_{\max}\right)\right]$$

(8) $$Cl=\frac{\mathrm{Dose}}{{\left(\mathrm{AUC}\right)}_{0-\infty }}$$

The corresponding values are listed in results.

### 2.7. Histopathology

A complete microscopic examination of the organs (liver, lung, kidneys, and spleen) from control animals (injected with PBS), and PEG-AuNPs-injected rats (after 24 h, 7 days, and 28 days) was performed. The formalin-fixed tissue samples were washed, dehydrated, and embedded in paraffin. Thereafter, 4 μm-thick sections were stained with hematoxylin and eosin. The tissue structure of particular organs was acquired on a Motic (Hong Kong, China) light microscope equipped with a MOTICAM 3+ color CCD camera using the MOTIC IMAGES PLUS 3.0 ML (Hong Kong, China) software. 

### 2.8. Hematological Analysis

Blood samples were collected using ethylenediamin tetra-acetic acid (EDTA) tubes at 1 h, 4 h, 24 h, and 28 days post-injection. Hematology analysis was performed using a hematological analyzer Sysmex K-4500, SYSMEX TOA Medical Electronics Co. LTD, Kobe, Japan. Parameters scheduled for examination were: leukocyte count, erythrocyte count, hemoglobin, hematocrit, mean corpuscular volume, mean corpuscular hemoglobin, mean corpuscular hemoglobin concentration, platelet count, percentage of lymphocytes, and lymphocyte count.

### 2.9. Clinical Chemistry

Before sacrifice, blood samples from the vena cava inferior were taken from all anesthetized animals for blood chemistry examination. Samples were analyzed using an Ortho Clinical Vitros^®^ 250 Chemistry System (Ortho-Clinical Diagnostics, Raritan, NJ, USA). Methodologies included colorimetric, potentiometric, and rate tests using multi-layered Vitros Slides. Blood samples were stored at room temperature (17–25 °C) for a maximum of 4 h until measurement. Parameters included: serum total protein (S-TP), serum albumin (S-Alb), serum urea (S-urea), serum creatinine (S-creat), serum cholesterol (S-chol), serum triglycerides (S-TAG), serum aspartate aminotransferase (S-AST, E.C. 2.6.1.1), serum alanine aminotransferase (S-ALT, E.C. 2.6.1.2), serum calcium (S-Ca), serum magnesium (S-Mg), and serum phosphorus (S-P).

### 2.10. The Genotoxic Effects of PEG-AuNPs 

#### 2.10.1. Mononuclear Blood Cells

The single-cell gel electrophoresis (SCGE also called the comet assay) was used to investigate the capacity of PEG-AuNPs to induce the DNA strand breaks (sb) in blood cells and kidneys at several time intervals (1 h, 4 h, 24 h, 7 days, and 28 days) after a single i.v. PEG-AuNPs injection. In control animals, the basal levels of sb in blood were evaluated only 1 h after PBS administration. This time-point interval was selected to avoid false-positive results in the case of PEG-AuNPs-injected animals as a consequence of the anesthesia. In brief, 30 µL of full blood was mixed with 600 µL LMP agarose (final concentration 0.75%) and 80 µL of cell suspension was immediately spread on a 1% NMP agarose pre-coated microscopic slides. After solidification of the gels, the slides were placed in lysis solution (2.5 M NaCl, 100 mM Na_2_EDTA, 10 mM Tris-HCl, pH 10 and 1% Triton X-100) for 1 h at 4 °C) to remove cellular proteins. After lysis, slides were transferred to an electrophoresis box and immersed in an alkaline solution (300 mM NaOH, 1 mM Na_2_EDTA, pH > 13). After 30 min of unwinding time, a voltage of 25 V (0.8 V/cm) was applied for 20 min at 4 °C. The slides were neutralized 3 × 5 min in Tris–HCl (0.4 M, pH 7.4). Before scoring, slides were stained with ethidium bromide (EtBr, 5 µg/mL). EtBr-stained nucleoids were examined with the Zeiss Axio Imager.Z2 fluorescence microscope (Zeiss, Germany) using the computerized image analysis (Metafer 3.6, MetaSystems GmbH, Altlussheim, Germany). The percentage of DNA in the tail (% of tail DNA) was used as a parameter for DNA damage measurement. One hundred comets were scored per sample in each gel.

#### 2.10.2. Primary Renal Cells

The left kidney was removed from the sacrificed rat, de-capsulated, bisected, and the medulla was removed. The cortex was chopped on ice using a scalpel and transferred into the tube with DMEM containing 1 mg/mL collagenase. The tissue was digested by incubating in collagenase at 37 °C for 7 min. The digested tissue was then filtered through the cell strainer (100 µg mesh size), washed with PBS, and centrifuged at 900× *g* for 10 min. The pellet was re-suspended in LMP agarose and assayed for the presence of DNA breakage by the comet assay as described above. 

### 2.11. Detection of Oxidative Damage to DNA 

After lysis, slides were washed three times for 5 min in endonuclease buffer (40 mM HEPES KOH, 0.1 M KCl, 0.5 mM EDTA, pH 8.0) and then incubated with formamidopyrimidine-DNA glycosylase/AP nuclease (Fpg, 0.2 U/slide) for 30 min in a humidified atmosphere at 37 °C. The slides were then transferred to an electrophoresis box and immersed in an alkaline solution. SCGE was then performed as described above. The level of oxidative DNA lesions identified at particular time-point intervals was expressed as a net Fpg value (i.e., the level of sb detected in DNA sample after incubation with Fpg enzyme minus the level of sb detected in the same DNA sample after incubation with buffer).

### 2.12. Statistical Analysis

The normality of data distribution was tested using the Sapiro–Wilk test. Statistical comparisons between groups were performed by the Mann–Whitney test, non-parametric testing for unrelated samples. Data were reported as the mean ± a standard error of the mean (SEM). The differences between control cells and treated cells in SCGE were evaluated by Student’s *t*-test. Obtained data were statistically analyzed using SPSS 23.0 software (Chicago, IL, USA) and GraphPad Prism 6.01 (La Jolla, CA, USA). The threshold of statistical significance was set to *p* < 0.05. 

## 3. Results

### 3.1. The Effect of PEG-AuNPs on Animal Body Weight

No visible signs of morbidity were observed in PEG-AuNPs-injected animals throughout the study. All animals showed a healthy appearance and normal activity without lethargy or apathy after PEG-AuNPs administration. During the follow-up study, the body weight increases of PEG-AuNPs-injected rats lagged slightly behind control animals (Table 2). This difference, however, did not reach statistical significance and represented approximately a 7% variance globally. 

### 3.2. Pharmacokinetic Analysis

**Figure 2:**
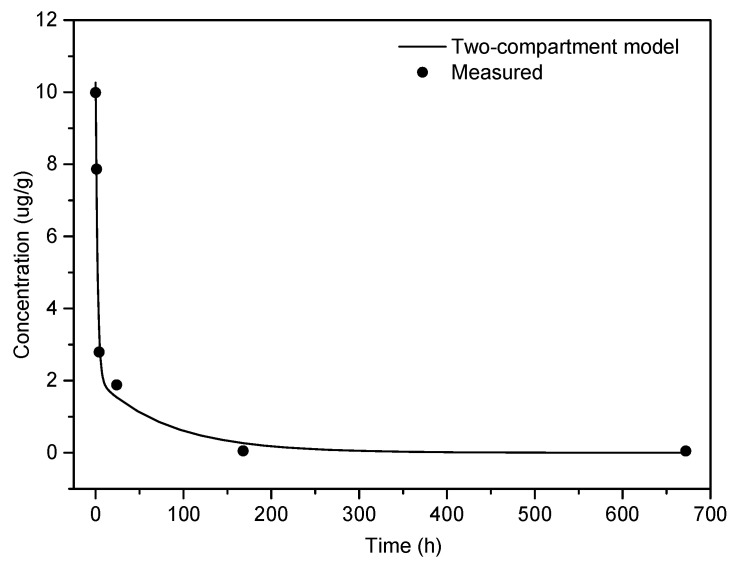
Time course of PEG-AuNPs blood concentration after a single dose i.v. administration (mean dose 160.5 µg). The solid line corresponds to the least-squares fit by the two-compartment model.

To determine the pharmacokinetic profile of PEG-AuNPs, the elemental Au content was assessed using GFAAS in whole blood and selected organs/tissues including liver, lungs, kidneys, and spleen at 1 h, 4 h, 24 h, 7 days, and 28 days after PEG-AuNPs injection. The gold detection in the organism is feasible because, under normal conditions, there is no background level in the tissues. The percentage of gold recovery in the analyzed samples detected 1 h after i.v. injection accounted for approximately 68.5% of the total injected dose. The kinetics of gold clearance from the bloodstream fits into a two-compartment pharmacokinetic model (Figure 2). The pharmacokinetic analysis revealed a rapid drop in the initial amount with a biodistribution half-life of 1.56 h; the distribution phase is followed by a slow, gradual elimination with a considerably longer half-life (57.0 h). This is also reflected in significantly longer mean residence time (MRT) for the whole body (74.6 h) compared to MRT in the blood (18.3 h). More than 80% of the PEG-AuNPs injected dose was eliminated from circulation within 24 h, but blood gold levels remained detectable (<0.6 %) even 28 days after administration (Figure 2). Other pharmacokinetic parameters, estimated by compartmental analysis, are summarized in Table 3. 

### 3.3. Tissue Distribution of PEG-AuNPs 

The gold was detected in all organs analyzed (liver, lung, spleen, kidney), indicating the widespread distribution of the PEG-AuNPs in the body. The gold content in blood and organs per gram of organ is presented in Table 4. One hour after injection, the highest gold concentration was determined in the blood, followed by lungs, spleen, liver, and kidneys, while 24 h after PEG-AuNPs administration, the highest gold concentration was quantified in the spleen, followed blood, liver, lungs, and kidneys. On day 7 and day 28 post-injection, the spleen followed by the liver were the organs with the highest level of gold concentration. The total gold recovery in organs at individual sampling times when compared with the administered dose is shown in Table 5. The highest gold content was found in the blood, followed by the liver and spleen at 24 h after PEG-AuNPs administration. On day 7 and day 28 post-injection, the highest quantity of gold was determined in the liver, followed by the spleen. Approximately similar amounts of gold were quantified in the liver at nearly each time interval, with a slight increase in its amount at day 7 and 28 after injection. The spleen was the second organ in terms of the amount of gold accumulation. A small amount of gold was also accumulated in the lungs and kidneys, and the kinetics of gold clearance from these two organs showed a time-dependent character. In contrast, the amount of elemental gold in the spleen increased gradually and peaked at 24 h after PEG-AuNPs injection; later on, gold content in this organ declined slowly. The sinusoidal pattern of gold accumulation in the spleen is closely linked with the kinetics of gold clearance from the blood, as the spleen is a center of the mononuclear phagocyte system removing pathogens/foreign particles from the blood circulation. In general, the liver and spleen were the preferential sites for gold accumulation in the rat body. However, given the size of the particular organ, PEG-AuNPs accumulated predominantly in the liver; the quantity of gold in the liver represented approximately 74% of the whole remaining amount of gold detected on day 7 and 28 after PEG-AuNPs administration. 

### 3.4. Histological Analysis of Tissues

**Figure 3:**
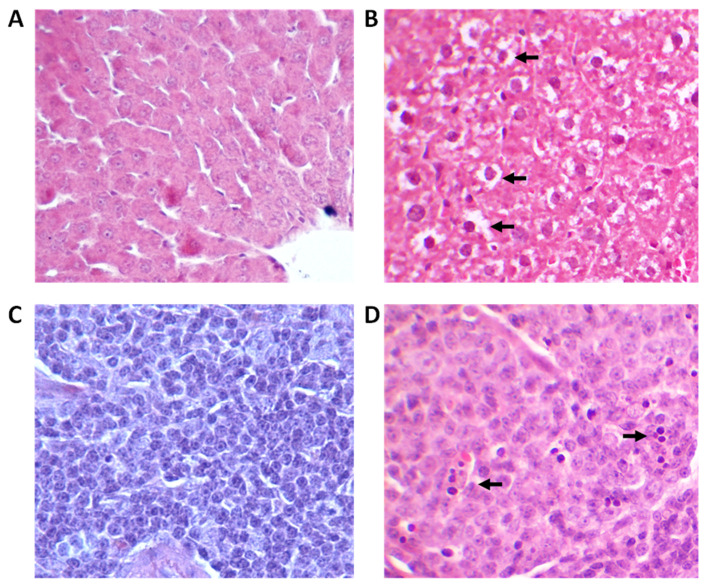
Histopathologic changes detected in rats exposed to the liver (**A**,**B**) and spleen (**C**,**D**). Cytoplasmic vacuolation (arrows) observed in rat hepatocytes at 7 days after single i.v. injection of PEG-AuNPs. Hematoxylin-eosin, magnification 20×. Shrinking of cells (arrows) in the splenic white pulp indicating apoptotic morphology observed in rat spleen 24 h after single i.v. injection of PEG-AuNPs. Hematoxylin-eosin, magnification 40×.

Macroscopic examination of lung, kidney, spleen, and liver tissue did not show any changes 24 h, 7 days, and 28 days after the PEG-AuNPs administration, as compared to organs of control rats. To explore in more detail the presence of tissue abnormalities induced by PEG-AuNPs, a complete microscopic histological examination of selected organs was performed using hematoxylin-eosin staining. No sign of inflammation or fibrosis was identified in lung and kidney tissues of exposed animals compared to the control group 24 h, 7 days, and 28 days after injection. On the other hand, increased cytoplasmic vacuolation (CV) was observed in the hepatocytes of nearly all PEG-AuNPs-injected animals 24 h and 7 days after exposure (Figure 3A,B). This lesion was, however, a transient one as no CV was found in the liver tissue on day 28 post-exposure. 

The presence of a small amount of intracytoplasmic golden-brown pigment granules within the splenic red pulp macrophages was observed in control as well as PEG-AuNPs-treated rats. This pigment is a common background finding in rodents and can be hemosiderin-, ceroid/lipofuscin-, or melanin-related; therefore, this lesion is not related to PEG-AuNPs exposure. Shrinking of cells in the white splenic pulp showing apoptotic morphology was observed in half of the rats 24 h after PEG-AuNPs administration (Figure 3C,D). Such histopathological lesion is very often detected in exposed animals as a physiological response of tissue to exposure and indicates an acute immune response to the presence of PEG-AuNPs. No such lesion was observed in the spleen 28 days after exposure.

### 3.5. Biochemical and Hematological Analysis on the Blood of Rats

The examination of the biochemical parameters in the blood serum can provide information about potential liver- and kidney-function impairment due to exposure. A significant increase in urea level determined in the serum of rats injected with PEG-AuNPs 4 h after treatment indicated poor glomerular blood filtration (Table 6). This deviation from the standard value was only transient because no elevated serum urea level was determined at later time intervals. It might be related to kidney overload due to PEG-AuNPs elimination from the blood. As the creatinine serum level was not affected during the whole study, one might suggest that increased urea is rather related to the liver impairment due to PEG-AuNPs exposure. Significant changes in AST and ALT, key markers of liver injury, were determined on day 28 after PEG-AuNPs-injection. While the level of ALT was decreased, the AST serum level was highly significantly elevated. Moreover, PEG-AuNPs also affected lipid metabolism. A significant increase in the TAG level was determined 4 h post-injection, while increased serum cholesterol level was estimated 24 h and 28 days after PEG-AuNPs administration (Table 6). 

Compared to the control group, changes in some hematological parameters were found in PEG-AuNPs-injected animals as well (Table 7). Surprisingly, an increased number of red blood cells and hemoglobin was detected 1 h, 4 h, and 28 days after PEG-AuNPs-injection, and a decreased platelets count was determined at 4 h, 24 h, and 28 days PEG-AuNPs post-injection. A decreased percentage of monocytes found 4 h and 24 h after exposure and an increased percentage of cells observed 28 days after exposure were statistically significant. We suppose, that differences in platelet count and percentage of monocytes reflect the normal variation and are not biologically relevant. 

### 3.6. Genotoxic Effects and Oxidative Damage to DNA Induced by PEG-AuNPs

**Figure 4:**
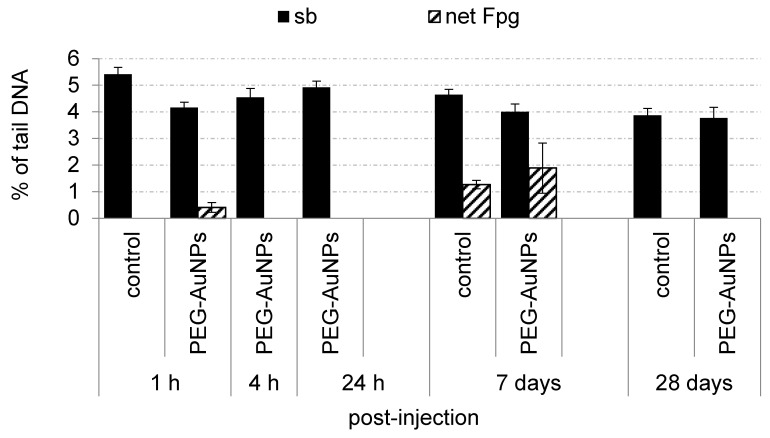
Genotoxic effects and oxidative damage to DNA induced by PEG-AuNPs in the blood mononuclear cells. Data are presented as mean ± standard error of the mean (SEM).

**Figure 5:**
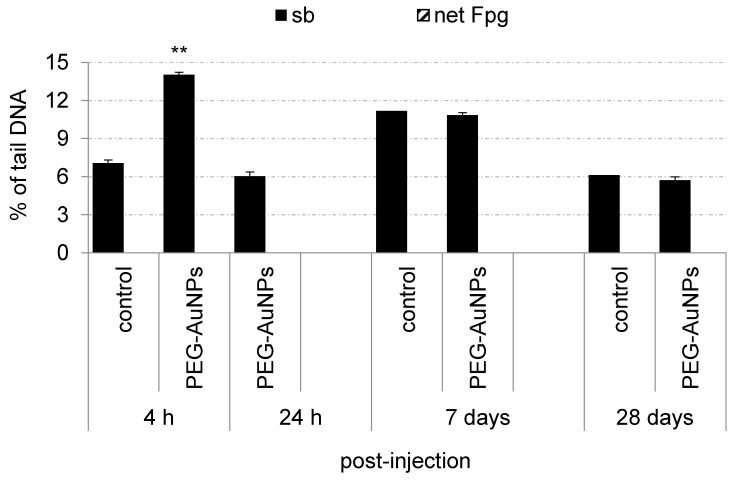
Genotoxic effects and oxidative damage to DNA induced by PEG-AuNPs in the primary renal cells.Data are presented as mean ± standard error of the mean (SEM). Significance: ** *p* < 0.01.

The impact of PEG-AuNPs on genome stability, including their capacity to induce oxidative DNA damage, was evaluated in the blood mononuclear cells and primary renal cells. PEG-AuNPs induced neither increased levels of DNA breakage nor oxidative DNA damage compared to the control group in blood cells during the whole study (Figure 4). In contrast, a significant rise of DNA strand breaks compared to control was determined at 4 h post-injection of PEG-AuNPs but not at other time intervals in primary renal cells (Figure 5). The basal levels of sb and oxidative DNA damage in control primary renal cells after short-term treatment were evaluated only at 1 h after PBS administration. This time-point interval was selected to avoid false-positive results in the case of PEG-AuNPs-injected animals as a consequence of the anesthesia. 

## 4. Discussion

Gold nanoparticles offer numerous opportunities for biomedical applications, especially in diagnosis and therapy. However, after systemic administration, AuNPs are redistributed throughout the body and accumulate not only in the target tissue but also in other vital organs and might produce toxic effects. Recent evidence of intralysosomal biodegradation of AuNPs, which over time leads to the formation of new bio-persistent nanostructures, raises concerns about the long-term effects of retained nanoparticles in the body [3]. Because the biodegradation process is very slow, it is necessary to perform a toxicological evaluation over a longer period to obtain a complex toxicological profile and a better understanding of the whole life cycle of AuNPs in the body [14]. Despite a large number of studies carried out during the past years, AuNPs toxicity remains fragmentary and even contradictory [15].

In our study, the effects of PEG-AuNPs were investigated up to 28 days after i.v. application of a single dose of 0.7 mg/kg to rats. Tail vein injection is an easily accessible procedure for animals, showing the lowest toxicity for AuNPs compared to oral or intraperitoneal administration [16]. Moreover, it mimics the most likely mode of nano-based drug applications in clinical practice. Rats were selected for this study as they are considered a more appropriate representative model of humans than mice in the animal-to-human extrapolation, based on most physiological parameters [8]. Spleen capillaries in rats and humans are sinusoidal, allowing large amounts of blood flow through open-loop pathways with AuNP filtration, while mouse spleen capillaries are non-sinusoidal [17]. Further, the average number of fenestrae per square micrometer of the mouse liver capillaries is lower than that of rats and humans [18]. Bahamonde et al. have recently reported a relatively higher accumulation of AuNPs in the spleen of rats compared to mice, and the development of granulomas in the mouse liver, while no such alterations were found in rats [19]. 

Pharmacokinetic (PK) characteristics and biodistribution data are essential for proper risk assessment of nanoparticles and largely define their therapeutic effect [20]. Physicochemical properties of nanoparticles, including size, surface charge, and surface chemistry, are important factors that determine these parameters. The surface of AuNPs (~13 nm) used in our in vivo experiment was coated with high molecular weight (5000 Da) PEG, an FDA approved polymer, which is commonly used in biomedical applications to improve the efficiency of drug and gene delivery to target cells and tissues [21]. PEGylation protects the particle surface from aggregation, increases their biocompatibility, and prolongs system circulation time. AuNPs coated with 5000 Da PEG have been reported to be more stable compared to AuNPs coated with low molecular weight (2000 Da) PEG [22] and less toxic [23]. Since nanoparticle PK is also dose-dependent, rats were injected with a dose of 0.7 mg/kg of body weight which correlates with the actual dose humans are exposed to [8]. The blood half-life (T_1/2β_) of our PEG-AuNPs was relatively long, comprising 57 h. The half-life of PEGylated AuNPs with similar physicochemical properties (13 nm, PEG 5 kDa) after single i.v. injection of 0.85 mg/kg in mice was estimated to 28.50 h [24]. Interestingly, T_1/2β_ of 15 nm PEG-AuNPs (PEG 5 kDa) in mice was ∼4.4 times greater (T_1/2β_ = 31.9 h) compared to 100 nm PEG-AuNPs (T_1/2β_ = 7.3 h) [25]. On the other hand, dextran-coated AuNPs (20 nm, 1 mg/kg), administered via a single i.v. injection to mice were rapidly eliminated from the blood circulation (T_1/2β_ = 5 h) [26]. Jo et al. studied PK of bare AuNPs (5–15 nm) after a single oral administration in rats [27]. The blood circulation half-life (T_1/2β_) of these AuNPs was 16.74 h. 

Although PEG-AuNPs were determined in all analyzed tissues, the highest quantity of gold was quantified in the liver and spleen. The spleen retained even more gold throughout the study when expressed as µg/g tissue. Our results are consistent with many other studies showing the highest levels of intravenously injected AuNPs in the liver and spleen where they remained for more than 2 months (reviewed in [7,8]). The sequestration of the AuNPs into the liver and spleen is rapid and occurs within minutes. The preferential accumulation of AuNPs in these organs is attributed to the fact that the liver and spleen are the major organs of the reticuloendothelial system with a high number of phagocytic cells and capillary beds. A small amount of gold was also found in the lungs and kidneys; the gold content in these organs gradually decreased over time. Renal clearance of nanoparticles through glomerular filtration is limited by nanoparticle size, thus particles above 6 nm usually cannot pass through the glomerular endothelial cells, and hence are not cleared [28]. However, in passing through the special architecture of peritubular renal capillaries, the particles could be partially eliminated via tubular reabsorption and transient translocation by the proximal tubular cells [29]. Low concentrations of gold were detected in the urine of both mice and rats up to 7 days post-exposure to 13 nm citrate- and PEG-coated (5000 MW) AuNPs after i.v. administration [19]. This data supported the assumption that even larger nanoparticles can be excreted through the kidneys following i.v. administration. Although lungs were not the primary organ of exposure, gold entered the lungs through deoxygenated blood from the tail vein. The presence of gold in the lungs after i.v. administration was also reported in several other studies [30,31,32]. 

Although macroscopic examination of lung, kidney, spleen, and liver tissue did not show any changes after the PEG-AuNPs administration, increased cytoplasmic vacuolation (CV) was observed in the hepatocytes of nearly all animals 24 h and 7 days after PEG-AuNPs administration. CV is a well-known morphological phenomenon observed in mammalian cells after exposure to pathogens as well as to various low-molecular-weight compounds [33]. Such lesions can be transient, observed only during the exposure to an inducer, or irreversible, often leading to cell death. Vacuolated swelling of hepatocytes was also observed in rats after intraperitoneal AuNPs administration [34]. Although no CV was detected at 28 days post-injections, we determined significant changes in the liver enzymes AST and ALT, the most sensitive markers for hepatic toxicity, albumin, and cholesterol at this time interval. Interestingly, along with the increase in AST serum level, we detected, a decrease in ALT serum level. A similar phenomenon was observed by Bednarski et al. in rats after oral administration of 25 nm AuNPs at 3 days post-treatment [35] and Abdehalim et al. [34] after intraperitoneal administration of 10 and 50 nm of AuNPs to rats during 3 days. A significant increase in AST and ALT enzymes was also determined in rats after repeated oral administration of 20 µg/kg of AuNPs (10–25 nm) during 21 days. Moreover, congestion around hepatocytes and in the liver parenchyma, a moderate degree of inflammation, small foci of necrosis, and binucleated cells were revealed by histopathological assessment as well [36]. On the other hand, no histopathological changes in the liver were assessed in rats after 14-day repeated oral administration of 1.3 mg/kg of AuNPs [27] or after a single i.v. administration of a dose of 1 mg/kg [19]. 

Shrinking of cells in the white splenic pulp indicating apoptotic morphology has been observed in two rats exposed to PEG-AuNPs after 24 h post-injection. The white pulp functions in producing and growing immune and blood cells, while the red pulp functions in filtering the blood of antigens, microorganisms, and defective or worn-out red blood cells. Terentyuk et al. showed that i.v. injection of 50 nm PEGylated AuNPs to rats can cause blood congestion in the red pulp and damage to the white pulp [37]. The gold nanoparticles (50 nm) were shown to primarily interact with B cells [38]. This data indicates that AuNPs could modulate the immune response in treated animals.

While no histopathological changes were observed in kidney cells, a significantly increased urea level was determined at 4 h post-injection indicating a poor glomerular blood filtration. Urea levels are useful indicators of renal status and kidney functionality. Nearly two-fold higher levels of urea were observed in rats after oral administration of AuNPs [36]. The change in this clinical chemistry parameter correlated with the increased DNA damage in primary renal cells detected by the comet assay. Because no renal disorders were detected at later intervals, we hypothesized that they could be transient, caused by a renal overload due to PEG-AuNP clearance from the blood.

The evaluation of the hemocompatibility is an important step during the biosafety screening of nanoparticles because the blood and blood components are the first potential target of interaction after i.v. administration. We found a significant increase in red blood cells (RBCs) and hemoglobin (HGB) levels 28 days after PEG-AuNPs administration. In contrast, Fraga et al. [12] detected a significant decrease in the number of RBCs, HGB, and hematocrit (HCT) compatible with a mild form of anemia at day 28 post-injection after single i.v. administration of ~20 nm citrate- and pentapeptide CALNN-coated AuNPs in rats. Interestingly, a size-dependent effect on hematological parameters following *i.p.* injection of 4 mg/kg of PEG-coated AuNPs in mice was observed. While 10 nm AuNPs induced an increase in white blood cells (WBCs), the 5 nm and 30 nm AuNPs induced a decrease in WBCs and RBCs at 28 days after injection [16]. It cannot be excluded that the surface chemistry of these AuNPs might substantially contribute to these differences in response.

## 5. Conclusions

PEG-AuNPs used in our study were relatively slowly cleared from the blood and accumulated in all selected organs, mostly in the liver and spleen. A small amount of PEG-AuNPs was detected even 28 days after a single dose administration. Histopathological changes determined in the liver and alterations in biochemical parameters (AST, ALT) indicated potential hepatotoxicity of these PEG-AuNPs. Although no histopathological changes were observed in the kidneys and could be interpreted as incidental and not exposure-related PEG-AuNPs could affect the renal function based on an increased level of serum urea and significant DNA breakage detected 4 h after injection. On the other hand, hematological parameters were not substantially influenced. To comprehensively assess the biosafety of gold nanoparticles, further studies focusing on their immunomodulatory effects as well as their ability to affect gene expression will be warranted.

## Figures and Tables

**Table 1:** Main physicochemical parameters of PEG-AuNPs.

	PEG-AuNPs
Core size [nm] ^a^	10.5 ± 0.83
Hydrodynamic size [nm] ^b^	13.1 ± 3.02
Surface modification	Polyethylene glycol (PEG)-Thiol, MW 5000 (Ω-end = SH, α-end = OCH)
PDI ^b^	0.2014 ± 0.019
Concentration: NPs/mL mmol/mL mg/ml	~4.05 × 10^14^ 2.01 × 10^−3^ 0.396
Zeta-potential [mV] ^b^	−9.12 ± 1.58
Solvent	LPS-free sterile MilliQ-water

^a^ Size was determined by transmission electron microscopy (TEM); ^b^ Size was determined by dynamic light scattering (DLS). Results are expressed as mean ± sd of 3 independent experiments.

**Table 2:** Bodyweight of control and PEG-AuNPs-injected rats.

Group	Initial Weight [g]	At 7 Days [g]	*p*- Value	At 28 Days [g]	*p*- Value
Control (PBS)	218.57 ± 11.00	247.50 ± 13.99	-	309.33 ± 15.50	-
PEG-AuNPs	229.28 ± 7.89	243.22 ± 19.19	0.644	288.33 ±18.06	0.122

**Table 3:** Pharmacokinetic parameters obtained from the two-compartment model fitted to PEG-AuNPs concentration in the blood. The values of corresponding composite parameters are *A* = (8.2 ± 1.3) μg g^−1^, *B* = (2.1 ± 1.2) μg g^−1^, *α* = (0.45 ± 0.16) h^−1^, *β* = (0.012 ± 0.017) h^−1^.

Parameter	Value
*t*_1/2α_ (biodistribution half-life)	1.56 h
*t*_1/2β_ (elimination half-life)	57.0 h
(AUC)_0–∞_	188 μg g^−1^ h
*t*_max_ (peripheral)	8.31 h
*C*_max_ (peripheral)	6.33 μg g^−1^
*Cl* (total body clearance)	1.09 mL h^−1^
*V*_d_ (initial volume of distribution)	19.9 mL

**Table 4:** Gold amount in blood and organs per gram of tissue determined by GFAAS in rats at different time intervals after a single i.v. injection of PEG-AuNPs (0.7 mg/kg).

Organ	Gold Concentration at Particular Time Intervals [µg/g]				
	1 h	4 h	24 h	7 Days	28 Days
Blood	7.866 ± 1.316	2.792 ± 0.836	1.882 ± 0.385	0.052 ± 0.005	0.047 ± 0.013
Liver	1.546 ± 0.415	1.113 ± 0.293	1.380 ± 0.251	2.223 ± 0.260	2.153 ± 0.361
Lungs	2.954 ± 0.915	1.529 ± 0.230	1.099 ± 0.290	0.718 ± 0.153	0.904 ± 0.159
Kidneys	1.421 ± 0.178	0.819 ± 0.196	0.453 ± 0.115	0.209 ± 0.067	0.272 ± 0.023
Spleen	2.693 ±0.273	4.024 ± 1.337	10.594 ± 1.116	5.695 ± 1.037	4.410 ± 1.408

**Table 5:** Gold content in blood and organs determined by GFAAS in rats at different time intervals after a single i.v. injection of PEG-AuNPs (0.7 mg/kg).

Organ	The gold Content in Blood and Organs at Particular Time Intervals [µg]				
	1 h	4 h	24 h	7 Days	28 Days
Blood	126.172 ± 27.011	44.779 ± 17.162	30.184 ± 7.909	0.889 ± 0.073	0.955 ± 0.186
Liver	7.973 ± 0.824	5.737 ± 0.497	7.115 ± 1.293	11.460 ± 1.338	11.100 ± 1.145
Lungs	3.214 ± 0.995	1.663 ± 0.250	1.195 ± 0.316	0.782 ± 0.167	0.984 ± 0.173
Kidneys	1.521 ± 0.178	1.068 ± 0.255	0.591 ± 0.149	0.272 ± 0.087	0.354 ± 0.030
Spleen	0.986 ± 0.100	1.473 ± 0.328	3.877 ± 0.408	2.085 ± 0.379	1.616 ± 0.515

**Table 6:** Biochemical parameters in rat blood at 1 h, 4 h, and 24 h post-injection of PEG-AuNPs.

Parameter	Control (n = 4)	PEG-AuNPs			Control	PEG-AuNPs
		1 h (n = 5)	4 h (n = 5)	24 h (n = 5)	28 Days (n = 8)	28 Days (n = 8)
S-ALT	0.86 ± 0.04	1.09 ± 0.12	0.82 ± 0.04	0.81 ± 0.08	1.01 ± 0.07	0.79 ± 0.03 ^+^
S-AST	1.79 ± 0.14	2.18 ± 0.15	1.91 ± 0.20	2.05 ± 0.20	1.66 ± 0.16	2.29 ± 0.12 **
S-Urea	6.92 ± 0.30	7.33 ± 0.39	8.80 ± 0.47 **	6.74 ± 0.20	7.19 ± 0.66	7.40 ± 0.43
S-Alb	32.73 ± 0. 96	36.06 ± 1.07	33.54 ± 0.56	34.30 ± 0.50	33.13 ± 0.45	35.66 ± 0.85 *
S-TP	63.50 ± 1.4	66.96 ± 1.53	63.28 ± 1.55	67.14 ± 1.01	58.36 ± 1.16	66.74 ± 1.05
S-Chol	1.54 ± 0.14	1.76 ± 0.17	1.75 ± 0.13	2.23 ± 0.25 *	1.41 ± 0.09	2.08 ± 0.18 *
S-TAG	0.66 ± 0.16	0.45 ± 0.06	1.22 ± 0.04 **	0.66 ± 0.13	0.88 ±0.15	0.52 ± 0.11 ^+^
S-Creat	21.18 ± 1.67	23.64 ± 2.31	25.68 ± 1.27	26.60 ± 1.78	30.09 ± 7.12	27.44 ± 1.21
S-Ca	2.45 ± 0.05	2.52 ± 0.03	2.56 ± 0.03	2.46 ± 0.05	2.52 ± 0.08	2.44 ± 0.02
S-Mg	0.91 ± 0.05	1.18 ± 0.05	1.00 ± 0.06	0.89 ± 0.04	1.07 ± 0.14	1.06 ± 0.02
S-P	3.41 ± 0.24	4.35 ± 0.29	3.71 ± 0.18	3.57 ± 0.07	2.78 ± 0.27	3.62 ±0.11

Data are presented as mean ± standard error of the mean (SEM). Significance: */+ *p* < 0.05; ** *p* < 0.01. S-ALT—serum alanine aminotransferase, S-AST—serum aspartate aminotransferase, S-Urea—serum urea, S-Alb—serum albumin, S-TP—serum total proteins, S-Chol—serum cholesterol, S-TAG—serum triglycerides, S-creat—serum creatinine, S-Ca—serum calcium, S-Mg—serum magnesium, S-P—serum phosphorus.

**Table 7:** Hematological parameters in rats at 1 h, 4 h, 24 h, and 28 days post-injection of PEG-AuNPs.

Parameter	Unit	Control	PEG-AuNPs			Control	PEG-AuNPs
		(n = 4)	1 h (n = 5)	4 h (n = 5)	24 h (n = 5)	28 Days (n = 8)	28 Days (n = 8)
WBC	10^9^/L	6.93 ± 0.28	7.26 ± 0.64	5.72 ± 0.64	9.68 ± 1.24	5.97 ± 0.36	6.61 ± 0.63
RBC	10^12^/L	7.99 ± 0.18	8.94 ± 0.18 *	9.01 ± 0.17 *	7.89 ± 0.29	7.90 ± 0.26	8.75 ± 0.25 *
HGB	g/dL	16.08 ± 0.23	17.08 ± 0.34	17.46 ± 0.26 *	15.66 ± 0.27	15.23 ± 0.43	17.28 ± 0.38 *
HCT	%	48.00 ± 0.81	52.28 ± 1.11	51.84 ± 1.04	47.18 ± 1.06	46.06 ± 1.38	50.93 ± 1.29
MCV	fL	60.13 ± 0.34	58.52 ± 1.11	57.54 ± 0.63	59.94 ± 0.99	58.36 ± 0.25	58.22 ± 0.45
MCH	pg	20.13 ± 0.17	19.10 ± 0.43	19.36 ± 0.24 ^+^	19.92 ± 0.54	19.29 ± 0.29	19.77 ± 0.25
MCHC	g/dL	33.50 ± 0.08	32.68 ± 0.30 ^+^	33.68 ± 0.37	33.22 ± 0.35	33.07 ± 0.39	33.96 ± 0.28
PLT	10^9^/L	850.8 ± 42.7	749.8 ± 123.8	728.2 ± 29.5 ^+^	777.6 ± 28.3 ^+^	726.6 ± 46.8	803.9 ± 39.9
LYM%	%	80.65 ± 1.72	82.58 ± 3.04	76.8 ± 1.94	75.96 ± 1.82	85.80 ± 1.07	82.72 ± 1.15
LYM	10^3^/μL	5.60 ± 0.33	6.04 ± 0.62	4.42 ± 0.57	7.40 ± 1.09	5.10 ± 0.28	5.44 ± 0.48
Differential WBC Count							
Lymphocytes	%	80.25 ± 3.62	80.20 ± 2.29	82.20 ± 4.50	81.30 ± 2.78	86.79 ± 1.88	82.72 ± 1.63
Neutrophils	%	17.63 ± 3.49	14.60 ± 2.02	16.00 ± 4.53	16.90 ± 2.79	12.00 ± 1.49	15.22 ± 1.50
Monocytes	%	1.25 ± 0.60	1.10 ± 0.36	1.10 ± 0.54	0.90 ± 0.37 ^+^	0.29 ± 0.10	1.00 ± 0.25 *
Eosinophils	%	0.88 ± 0.38	0.60 ± 0.19	0.70 ± 0.26	0.90 ± 0.29	0.93 ± 0.53	1.06 ± 0.21
Basophils	%	0.00 ± 0.00	0.00 ± 0.00	0.00 ± 0.00	0.00 ± 0.00	0.00 ± 0.00	0.00 ± 0.00

Results are expressed as mean ± SEM. Significance: */+ *p* < 0.05. WBC—white blood cells; RBC—red blood cells; HGB—hemoglobin; HCT—hematocrit; MCV—mean cell volume; MCH—mean cell hemoglobin; MCHC—mean cell hemoglobin concentration; PLT—platelets; LYM—lymphocytes.

## Data Availability

Data presented in this article are available at request from the corresponding author.
